# Tunable two-phonon higher-order sideband amplification in a quadratically coupled optomechanical system

**DOI:** 10.1038/s41598-017-17974-y

**Published:** 2017-12-15

**Authors:** Shaopeng Liu, Wen-Xing Yang, Tao Shui, Zhonghu Zhu, Ai-Xi Chen

**Affiliations:** 1 Department of Physics, Southeast University, Nanjing, 211189 China; 2 Department of Physics, Zhejiang Sci-Tech University, Hangzhou, 310018 China; 3 Institute for Quantum Computing, University of Waterloo, Ontario, N2L 3G1 Canada

## Abstract

We propose an efficient scheme for the controllable amplification of two-phonon higher-order sidebands in a quadratically coupled optomechanical system. In this scheme, a strong control field and a weak probe pulse are injected into the cavity, and the membrane located at the middle position of the cavity is driven resonantly by a weak coherent mechanical pump. Beyond the conventional linearized approximation, we derive analytical expressions for the output transmission of probe pulse and the amplitude of second-order sideband by adding the nonlinear coefficients into the Heisenberg-Langevin formalism. Using experimentally achievable parameters, we identify the conditions under which the mechanical pump and the frequency detuning of control field allow us to modify the transmission of probe pulse and improve the amplitude of two-phonon higher-order sideband generation beyond what is achievable in absence of the mechanical pump. Furthermore, we also find that the higher-order sideband generation depends sensitively on the phase of mechanical pump when the control field becomes strong. The present proposal offers a practical opportunity to design chip-scale optical communications and optical frequency combs.

## Introduction

Cavity optomechanics^1^, that combines the optical degree of freedom with the mechanical degree of freedom via a radiation-pressure force, has experienced considerable achievements in linear optomechanical coupling regime, such as optomechanically induced transparency (OMIT)^2–4^, sideband cooling of mechanical resonator^5–7^ and normal-mode splitting^8,9^. As an extension of the investigation for nonlinear effects in microcavity system^10–12^, a variety of nonlinear optical phenomena^13–21^ including optical frequency comb^13^, optomechanical chaos^14,15^ and higher-order sideband generation^16–20^ have been studied theoretically and experimentally in the linear optomechanical system. In particular, it is of interest to explore higher-order sidebands that is used for the parametric frequency-conversion and nonlinear quantum nature in cavity optomechanics.

Recently, a new type of dispersive optomechanical device featuring quadratic optomechanical coupling has been exploited in the high-finesse Fabry-Pérot cavity^22–24^, where a flexible dielectric membrane locates at a node or antinode of the intracavity standing wave. In comparison with the standard linear optomechanical coupling, there are two outstanding advantages in such a quadratic optomechanical system. Firstly, the optical cavity field is proportional to the square of displacement or the phonon number of the membrane, so that this quadratic optomechanical coupling allows to implement a quantum non-demolition readout of the membrane’s energy eigenstate^23^. Secondly, the quadratic optomechanical coupling indicates two-phonon processes, which could provide a more accessible multiphonon sideband effect. In analogy with linear optomechanical symtem, this quadratic optomechanical coupling has also been extended to numerous studies, such as the two-phonon OMIT^25,26^ and amplification^24,27^, cooling and squeezing of the mechanical oscillator^28–32^, and the preparation of quantum superposition states^33–35^. Taking these advantages of quadratic coupling into consideration, a promising route for the two-phonon higher-order sideband generation seems to be established in this quadratically coupled optomechanical system.

In this paper, we demonstrate that a quadratically coupled optomechanical system is suggested to provide a controllable multiphonon sideband amplification. Although a few groups discussed the features of the output field in the quadratically coupled optomechanical system, they mainly concentrated on probe absorption spectrum or linearized phonon cooling based on the conventional linearized approximation^25–32^. Different from these general linear photon-phonon interaction, our scheme involving both nonlinear photon-phonon and multiphonon interactions is dependent upon the dynamical backaction of quadratic optomechanical coupling. Beyond the conventional linearized approximation, we derive analytical expressions for the output transmission of probe pulse and the amplitude of second-order sideband by adding nonlinear terms into the Heisenberg-Langevin equations. Furthermore, our results illustrate that the sideband amplification and phase-dependent effect in generated two-phonon higher-order sideband signals can be modulated by means of the amplitude and phase of the external mechanical pump.

## Theoretical model and basic equations of higher-order sidebands

**Figure 1:**
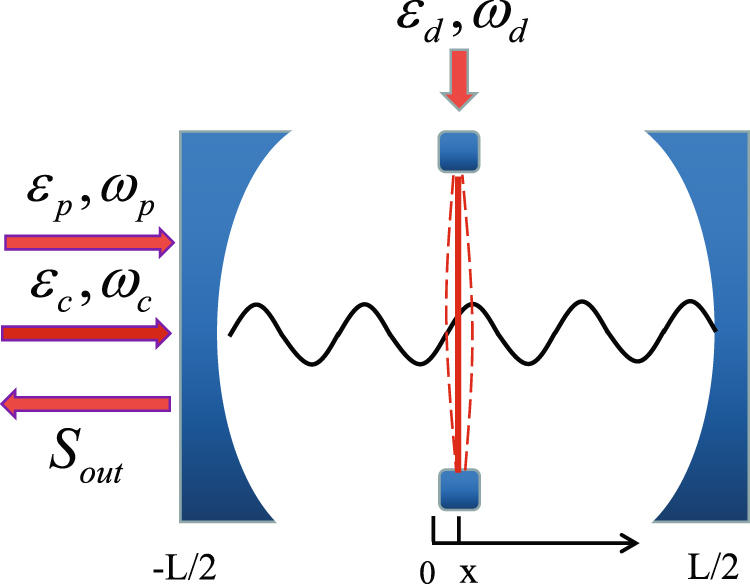
Schematic diagram of a quadratically coupled optomechanical system. This optomechanical system is driven by a strong control field (with frequency *ω*_*c*_) and a relatively weak probe pulse (with frequency *ω*_*p*_), while the thin dielectric membrane located at an antinode of the cavity field is excited by a weak coherent mechanical pump (with frequency *ω*_*d*_). After the dynamical backaction of quadratic optomechanical coupling between the cavity field and the membrane, the higher-order sidebands are generated.

As schematically shown in Fig. 1, a quadratic optomehcanical (or named dispersive optomehcanical) set-up, where the thin dielectric membrane with angular frequency *ω*_*m*_, effective mass *m* and finite reflectivity *R* is located at an antinode of the intracavity field, consists two fixed high-finesse mirrors separated from each other by a distance *L*. When this quadratically coupled optomechanical system is driven by the input field ${S}_{in}={\varepsilon }_{c}{e}^{-i{\omega }_{c}t-i{{\varphi }}_{c}}+{\varepsilon }_{p}{e}^{-i{\omega }_{p}t-i{{\varphi }}_{p}}$ (*ε*_*c*,*p*_, *ω*_*c*,*p*_ and *ϕ*_*c*,*p*_ denote the amplitude, center frequency and phase of the control field and probe pulse), a radiation-pressure force acts on the movable membrane and produces quadratic coupling constant $G=\frac{1}{2}\frac{{d}^{2}\omega }{d{x}^{2}}{|}_{x=0}\,=\,\frac{8{\pi }^{2}c}{L{\lambda }_{c}^{2}}\sqrt{\frac{R}{1-R}}$ ^25,29^ with the speed of light *c* in a vacuum and the wavelength of control field *λ*_*c*_. Simultaneously, under the condition of two-phonon resonance, a coherent mechanical pump (with amplitude *ε*_*d*_, center frequency *ω*_*d*_ and phase *ϕ*_*d*_) applied to the membrane is expected for creating a parametric amplification of mechanical mode^27,36^. Thus, this distinct membrane-in-the-middle configuration efficiently avoids compromising either the optical or mechanical functionality^22,23^. Under the approximation of quadratic optomechanical coupling, we begin our analysis by writing system’s Hamiltonian of this dispersive optomechanical cavity,

1 $$\begin{array}{ccc}H & = & \frac{{\hat{p}}^{2}}{2m}+\frac{1}{2}m{\omega }_{m}{\hat{x}}^{2}+i\hslash {\varepsilon }_{d}[({\hat{b}}^{\dagger }{)}^{2}{e}^{-i{\omega }_{d}t-i{{\varphi }}_{d}}-{\hat{b}}^{2}{e}^{i{\omega }_{d}t+i{{\varphi }}_{d}}]+\hslash G{\hat{a}}^{\dagger }\hat{a}{x}^{2}+\hslash {\omega }_{0}{\hat{a}}^{\dagger }\hat{a}\\  &  & +\,i\hslash \sqrt{{\eta }_{L}\kappa }{\varepsilon }_{c}({\hat{a}}^{\dagger }{e}^{-i{\omega }_{c}t}-\hat{a}{e}^{i{\omega }_{c}t})+i\hslash \sqrt{{\eta }_{L}\kappa }{\varepsilon }_{p}({\hat{a}}^{\dagger }{e}^{-i{\omega }_{p}t-i{{\varphi }}_{pc}}-\hat{a}{e}^{i{\omega }_{p}t+i{{\varphi }}_{pc}}),\end{array}$$

where $\hat{x}$ and $\hat{p}$ are the position and momentum operators of the membrane. $\hat{a}$ $({\hat{a}}^{\dagger })$ represents annihilation (creation) operator of the cavity mode with an unperturbed resonance frequency *ω*_0_. The fundamental membrane vibrational mode $\hat{b}$ $({\hat{b}}^{\dagger })$ comes from the quantization for the position and momentum operators of the membrane and is described via a relationship of $\hat{b}={({\hat{b}}^{\dagger })}^{\dagger }=\sqrt{m{\omega }_{m}/(2\hslash )}[\hat{x}+i\hat{p}/(m{\omega }_{m})]$. *ϕ*_*pc*_ = *ϕ*_*p*_ − *ϕ*_*c*_ is the relative phase between control field and probe pulse. In addition, the amplitudes *ε*_*c*,*p*_ of control field and probe pulse can be normalized to a photon flux at the input of the cavity^2^, i.e., ${\varepsilon }_{c,p}=\sqrt{{P}_{c,p}/\hslash {\omega }_{c,p}}$ with control field and probe pulse powers *P*_*c*,*p*_. The total loss rate is given by *κ* = *κ*_0_ + *κ*_*L*_ + *κ*_*R*_ with an intrinsic loss rate *κ*_0_ and an external loss rate of left (right) mirror *κ*_*L*_ = *η*_*L*_ *κ* (*κ*_*R*_ = *η*_*R*_ *κ*), where the coupling parameter *η*_*L*,*R*_ can be continuously adjusted by tuning the taper-resonator gap^37,38^.

In a rotating frame at the frequency of control field *ω*_*c*_, by substituting the expression of fundamental membrane vibrational mode into above original Hamiltonian, the interaction Hamiltonian can be obtained as

2 $$\begin{array}{ccc}{H}_{{int}} & = & \frac{{\hat{p}}^{2}}{2m}+\frac{1}{2}m{\omega }_{m}^{2}{\hat{x}}^{2}+\hslash G{\hat{a}}^{\dagger }\hat{a}{\hat{x}}^{2}+i\hslash \sqrt{{\eta }_{L}\kappa }{\varepsilon }_{c}({\hat{a}}^{\dagger }-\hat{a})+\hslash {{\rm{\Delta }}}_{c}{\hat{a}}^{\dagger }\hat{a}\\  &  & +i\hslash \sqrt{{\eta }_{L}\kappa }{\varepsilon }_{p}({\hat{a}}^{\dagger }{e}^{-i{{\rm{\Delta }}}_{p}t-i{\varphi }_{pc}}-\hat{a}{e}^{i{{\rm{\Delta }}}_{p}t+i{\varphi }_{pc}})\\  &  & +\,i\frac{m{\omega }_{m}}{2}{\varepsilon }_{d}[{(\hat{x}-i\frac{\hat{p}}{m{\omega }_{m}})}^{2}{e}^{-i{\omega }_{d}t-i{\varphi }_{d}}-{(\hat{x}+i\frac{\hat{p}}{m{\omega }_{m}})}^{2}{e}^{i{\omega }_{d}t+i{\varphi }_{d}}],\end{array}$$

here the corresponding frequency detunings are defined as Δ_*c*_ = *ω*_0_ − *ω*_*c*_ and Δ_*p*_ = *ω*_*p*_ − *ω*_*c*_. In our proposed quadratically coupled optomechanical system, the control field detuning Δ_*c*_ should be close to 2*ω*_*m*_, which satisfies the condition of two-phonon resonance. Then, in order to fully describe the motion dynamics of this quadratically coupled optomechanical cavity, the cavity damping and the dissipation process should be considered. By employing some shorthand definitions for the Heisenberg operators, i.e., $\hat{X}={\hat{x}}^{2}$, $\hat{P}={\hat{p}}^{2}$ and $\hat{Q}=\hat{x}\hat{p}+\hat{p}\hat{x}$, one readily gives the following Heisenberg-Langevin equations:

3 $${\partial }_{t}\hat{a}=-[\frac{\kappa }{2}+i({{\rm{\Delta }}}_{c}+G\hat{X})]\hat{a}+\sqrt{{\eta }_{L}\kappa }({\varepsilon }_{c}+{\varepsilon }_{p}{e}^{-i{{\rm{\Delta }}}_{p}t-i{{\varphi }}_{pc}})+\sqrt{(1-{\eta }_{L})\kappa }{\hat{a}}_{in},$$

4 $${\partial }_{t}\hat{x}=\frac{\hat{p}}{m}+\hat{x}{\varepsilon }_{d}({e}^{-i{\omega }_{d}t-i{{\varphi }}_{d}}+{e}^{i{\omega }_{d}t+i{{\varphi }}_{d}})-i\frac{\hat{p}}{m{\omega }_{m}}{\varepsilon }_{d}({e}^{-i{\omega }_{d}t-i{{\varphi }}_{d}}-{e}^{i{\omega }_{d}t+i{{\varphi }}_{d}}),$$

5 $$\begin{array}{ccc}{\partial }_{t}\hat{p} & = & -m{\omega }_{m}^{2}\hat{x}-{\Gamma }_{m}\hat{p}-2\hslash G{\hat{a}}^{\dagger }\hat{a}\hat{x}-im{\omega }_{m}\hat{x}{\varepsilon }_{d}({e}^{-i{\omega }_{d}t-i{\varphi }_{d}}-{e}^{i{\omega }_{d}t+i{\varphi }_{d}})\\  &  & -\,\hat{p}{\varepsilon }_{d}({e}^{-i{\omega }_{d}t-i{\varphi }_{d}}+{e}^{i{\omega }_{d}t+i{\varphi }_{d}})+{\hat{F}}_{th},\end{array}$$

6 $${\partial }_{t}\hat{X}=\frac{\hat{Q}}{m}-i\frac{\hat{Q}}{m{\omega }_{m}}{\varepsilon }_{d}({e}^{-i{\omega }_{d}t-i{\varphi }_{d}}-{e}^{i{\omega }_{d}t+i{\varphi }_{d}})+2\hat{X}{\varepsilon }_{d}({e}^{-i{\omega }_{d}t-i{\varphi }_{d}}+{e}^{i{\omega }_{d}t+i{\varphi }_{d}}),$$

7 $$\begin{array}{ccc}{\partial }_{t}\hat{P} & = & -(2\hslash G{\hat{a}}^{\dagger }\hat{a}+m{\omega }_{m}^{2})\hat{Q}-im{\omega }_{m}\hat{Q}{\varepsilon }_{d}({e}^{-i{\omega }_{d}t-i{\varphi }_{d}}-{e}^{i{\omega }_{d}t+i{\varphi }_{d}})-2\hat{P}{\varepsilon }_{d}({e}^{-i{\omega }_{d}t-i{\varphi }_{d}}+{e}^{i{\omega }_{d}t+i{\varphi }_{d}})\\  &  & +\,{\Gamma }_{m}(1+2{n}_{th})\hslash m{\omega }_{m}-2{\Gamma }_{m}\hat{P},\end{array}$$

8 $$\begin{array}{ccc}{\partial }_{t}\hat{Q} & = & -(4\hslash G{\hat{a}}^{\dagger }\hat{a}+2m{\omega }_{m}^{2})\hat{X}-{\Gamma }_{m}\hat{Q}+\frac{2\hat{P}}{m}-2im{\omega }_{m}\hat{X}{\varepsilon }_{d}({e}^{-i{\omega }_{d}t-i{\varphi }_{d}}-{e}^{i{\omega }_{d}t+i{\varphi }_{d}})\\  &  & -\,\frac{2i\hat{P}}{m{\omega }_{m}}{\varepsilon }_{d}({e}^{-i{\omega }_{d}t-i{\varphi }_{d}}-{e}^{i{\omega }_{d}t+i{\varphi }_{d}}),\end{array}$$

where the decay rate *κ* of cavity mode and the damping Γ_*m*_ of mechanical mode are phenomenologically added in above equations. The input vacuum noise operator is ${\hat{a}}_{in}$ with zero expectation value $\langle {\hat{a}}_{in}(t)\rangle =0$ and nonzero correlation function $\langle {\hat{a}}_{in}(t){\hat{a}}_{in}^{\dagger }(t^{\prime} )\rangle =\delta (t-t^{\prime} )$, while the thermal bath ${\hat{F}}_{th}$ of mechanical mode is affected by a Brownian stochastic force and governed by zero expectation value $\langle {\hat{F}}_{th}(t)\rangle =0$ and correlation function $\langle {\hat{F}}_{th}(t){\hat{F}}_{th}(t^{\prime} )\rangle =\frac{\hslash {{\rm{\Gamma }}}_{m}m}{2\pi }\int \omega {e}^{-i\omega (t-t^{\prime} )}[1+\,\coth (\frac{\hslash \omega }{2{k}_{B}T})]d\omega$ with the Boltzmann constant *k*_*B*_^39^. Owing to the existence of system’s thermal equilibrium at temperature *T*, the constant ${n}_{th}={[\exp (\hslash {\omega }_{m}/{k}_{B}T)-1]}^{-1}$ represents the mean thermal phonon number as a result of the coupling between the membrane and the thermal environment.

Except for the approximation of quadratic optomechanical coupling used in the system’s Hamiltonian, we still need to adopt three assumptions to study the multiphonon sideband effect, including the perturbation method^2,16^, the sideband-resolved limit (i.e., ${\omega }_{m}\gg \kappa$) and the factorization assumption of 〈*ab*〉 = 〈*a*〉〈*b*〉. Thus all of the operators can be expressed as a perturbation form of ${\mathscr{O}}(t)={{\mathscr{O}}}_{0}+\delta {\mathscr{O}}(t)$, where the operators are reduced to their expectation values, i.e., $a(t)\equiv \langle \hat{a}(t)\rangle$, $X(t)\equiv \langle {\hat{x}}^{2}(t)\rangle$, $P(t)\equiv \langle {\hat{p}}^{2}(t)\rangle$ and $Q(t)\equiv \langle \hat{x}(t)\hat{p}(t)+\hat{p}(t)\hat{x}(t)\rangle$. Note that the expectation values of noise operators including $\langle {\hat{a}}_{in}(t)\rangle$ and $\langle {\hat{F}}_{th}(t)\rangle$ have zero mean value. By taking these perturbation expressions into the Heisenberg-Langevin Eqs (3–8), we can obtain the steady-state solutions as

9 $${a}_{0}=\frac{\sqrt{{\eta }_{L}\kappa }{\varepsilon }_{c}}{\frac{\kappa }{2}+i{\bar{{\rm{\Delta }}}}_{c}},$$

10 $${X}_{0}=\frac{{P}_{0}}{{m}^{2}{\omega }_{m}^{2}(1+2\alpha )},$$

11 $${P}_{0}=(1+2{n}_{th})\frac{\hslash m{\omega }_{m}}{2},$$

12 $${Q}_{0}=0,$$

with the detuning of effective cavity resonance frequency ${\bar{{\rm{\Delta }}}}_{c}={{\rm{\Delta }}}_{c}+G{X}_{0}$ and the parameter $\alpha =\frac{\hslash G{|{a}_{0}|}^{2}}{m{\omega }_{m}^{2}}$.

Usually, the conventional linearized approximation in the quadratically coupled optomechanical system is used to deal with the steady-state solution or probe transmission by calculating the linearized Heisenberg-Langevin equations^25–32^. But a comprehensive treatment of the perturbation technique should include both linear terms ${{\mathscr{O}}}_{0}$ and nonlinear terms *δa δX*, *δa δQ*, *δa* ^*^ *δa*, *δa* ^*^ *δa δX* and *δa* ^*^ *δa δQ*, in which the linear terms support the steady-state theory while the nonlinear terms directly contribute to the generation of higher-order sidebands. A further analysis beyond the conventional linearized approximation is based on the transformations of these perturbation terms by setting the following ansatz:

13 $$\delta a=\sum _{n}({A}_{n}^{-}{e}^{-ni{{\rm{\Delta }}}_{p}t}+{A}_{n}^{+}{e}^{ni{{\rm{\Delta }}}_{p}t}),$$

14 $$\delta O=\sum _{n}({O}_{n}{e}^{-ni{{\rm{\Delta }}}_{p}t}+{O}_{n}^{\ast }{e}^{ni{{\rm{\Delta }}}_{p}t}),$$

with the *n* ^*th*^-order upper sideband ${A}_{n}^{-}$ and lower sideband ${A}_{n}^{+}$. The perturbation term $\delta {\mathscr{O}}$ in Eq. (14) represents *δX*, *δP* and *δQ*. Additionally, we assume *ω*_*d*_ = Δ_*p*_ so that the mechanical pump field has an effective influence on the generation of higher-order sidebands. From the form, above ansatz indicates the generated output fields with a series of frequency components (i.e., *ω*_*c*_ ± *n*Δ_*p*_ with the integer *n* being the order of sideband). For example, anti-Stokes field and Stokes field are the first upper sideband for *ω*_*d*_ + Δ_*p*_ and the first lower sideband for *ω*_*c*_ − Δ_*p*_, respectively. The output field with a new frequency *ω*_*d*_ + *n*Δ_*p*_ (*ω*_*c*_ − *n*Δ_*p*_) refers to the *n* ^*th*^-order upper (lower) sideband. Since the multi-photon optical processes are theoretically weaker than the linear optical process, we neglect higher order terms in the calculation of the lower order sideband. That is also the reason that perturbation terms can be ignored in the steady-state solution. By inserting Eqs (13) and (14) into Eqs (3–8) and comparing the coefficients of the same order, one can obtain several new equation set:

15 $${f}_{1}(n{{\rm{\Delta }}}_{p}){A}_{n}^{-}=-iG[{X}_{n}{a}_{0}+\sum _{z=1}^{n-1}({X}_{n-z}{A}_{z}^{-})]+\sqrt{{\eta }_{L}\kappa }{\delta }_{n,1}{\varepsilon }_{p}{e}^{-i{\varphi }_{pc}},$$

16 $${f}_{2}(n{{\rm{\Delta }}}_{p}){({A}_{n}^{+})}^{\ast }=iG\{{X}_{n}{a}_{0}^{\ast }+\sum _{z=1}^{n-1}[{X}_{n-z}{({A}_{z}^{+})}^{\ast }]\},$$

17 $$-in{{\rm{\Delta }}}_{p}{X}_{n}=\frac{{Q}_{n}}{m}+2({X}_{n-1}-i\frac{{Q}_{n-1}}{2m{\omega }_{m}}){\varepsilon }_{d}{e}^{-i{\varphi }_{d}},$$

18 $${f}_{3}(n{{\rm{\Delta }}}_{p}){P}_{n}=in{{\rm{\Delta }}}_{p}{m}^{2}{\omega }_{m}^{2}(1+2\alpha ){X}_{n}-{y}_{n}^{b}m,$$

19 $$m{f}_{4}(n{{\rm{\Delta }}}_{p}){X}_{n}=-4\hslash G[{({A}_{n}^{+})}^{\ast }{a}_{0}{X}_{0}+{a}_{0}^{\ast }{A}_{n}^{-}{X}_{0}]+\frac{2{P}_{n}}{m}+{y}_{n}^{c}m,$$

with

$$\begin{array}{rcl}{f}_{1}(n{{\rm{\Delta }}}_{p}) & = & \frac{\kappa }{2}+i{\bar{{\rm{\Delta }}}}_{c}-in{{\rm{\Delta }}}_{p},\\ {f}_{2}(n{{\rm{\Delta }}}_{p}) & = & \frac{\kappa }{2}-i{\bar{{\rm{\Delta }}}}_{c}-in{{\rm{\Delta }}}_{p},\\ {f}_{3}(n{{\rm{\Delta }}}_{p}) & = & 2{{\rm{\Gamma }}}_{m}-in{{\rm{\Delta }}}_{p},\\ {f}_{4}(n{{\rm{\Delta }}}_{p}) & = & (4\alpha +2){\omega }_{m}^{2}-in{{\rm{\Delta }}}_{p}({{\rm{\Gamma }}}_{m}-in{{\rm{\Delta }}}_{p}),\\ \zeta (n{{\rm{\Delta }}}_{p}) & = & {f}_{3}(n{{\rm{\Delta }}}_{p}){f}_{4}(n{{\rm{\Delta }}}_{p})-i(4\alpha +2)n{{\rm{\Delta }}}_{p}{\omega }_{m}^{2},\\ D(n{{\rm{\Delta }}}_{p}) & = & -8\bar{{\rm{\Delta }}}g\alpha {\omega }_{m}^{2}{X}_{0}\frac{{f}_{3}(n{{\rm{\Delta }}}_{p})}{{f}_{2}(n{{\rm{\Delta }}}_{p})}+{f}_{1}(n{{\rm{\Delta }}}_{p})\zeta (n{{\rm{\Delta }}}_{p}),\\ {y}_{n}^{a} & = & {X}_{n-1}-i\frac{{Q}_{n-1}}{2m{\omega }_{m}},\\ {y}_{n}^{b} & = & -\frac{2\hslash G}{m}\sum _{i,j,k}[{({A}_{i}^{+})}^{\ast }{A}_{j}^{-}{Q}_{k}]+[\frac{2{P}_{n-1}}{m}+i{\omega }_{m}{Q}_{n-1}-m{\omega }_{m}^{2}(4\alpha +2){y}_{n}^{a}]{\varepsilon }_{d}{e}^{-i{\varphi }_{d}},\\ {y}_{n}^{c} & = & -\frac{4\hslash G}{m}\sum _{i,j,k}[{({A}_{i}^{+})}^{\ast }{A}_{j}^{-}{X}_{k}]+[2({{\rm{\Gamma }}}_{m}-in{{\rm{\Delta }}}_{p}){y}_{n}^{a}-2i{\omega }_{m}{X}_{n-1}-2i\frac{{P}_{n-1}}{{m}^{2}{\omega }_{m}}]{\varepsilon }_{d}{e}^{-i{\varphi }_{d}},\end{array}$$

here the positive integers (*i*,*j*,*k*) satisfy *i* + *j* + *k* = *n* and *i*, *j*, *k* < *n*.

Then, we can have the analytical solutions for amplitudes of the first-order sidebands and the second-order sideband:

20 $$\begin{array}{rcl}{A}_{1}^{-} & = & \frac{1}{D({{\rm{\Delta }}}_{p})}\{[4i\alpha g{X}_{0}{\omega }_{m}^{2}\frac{{f}_{3}({{\rm{\Delta }}}_{p})}{{f}_{2}({{\rm{\Delta }}}_{p})}+\zeta ({{\rm{\Delta }}}_{p})]\sqrt{{\eta }_{L}\kappa }{\varepsilon }_{p}{e}^{-i{\varphi }_{pc}}-2ig{a}_{0}{X}_{0}{f}_{3}({{\rm{\Delta }}}_{p}){f}_{5}({{\rm{\Delta }}}_{p}){\varepsilon }_{d}{e}^{-i{\varphi }_{d}}\},\end{array}$$

21 $$\begin{array}{rcl}{A}_{1}^{+} & = & \frac{1}{{D}^{\ast }({{\rm{\Delta }}}_{p})}\{4i{g}^{2}\hslash {a}_{0}^{2}{X}_{0}\frac{{f}_{3}^{\ast }({{\rm{\Delta }}}_{p})}{m{f}_{2}^{\ast }({{\rm{\Delta }}}_{p})}\sqrt{{\eta }_{L}\kappa }{\varepsilon }_{p}{e}^{-i{\varphi }_{pc}}\\  &  & -\,2ig{a}_{0}{X}_{0}{f}_{1}^{\ast }({{\rm{\Delta }}}_{p}){f}_{5}^{\ast }({{\rm{\Delta }}}_{p})\frac{{f}_{3}^{\ast }({{\rm{\Delta }}}_{p})}{{f}_{2}^{\ast }({{\rm{\Delta }}}_{p})}{\varepsilon }_{d}{e}^{-i{\varphi }_{d}}\}\end{array},$$

22 $$\begin{array}{rcl}{A}_{2}^{-} & = & \frac{1}{D(2{{\rm{\Delta }}}_{p})}\{\frac{2i{a}_{0}g{y}_{2}^{b}}{m}-i{a}_{0}g{f}_{3}(2{{\rm{\Delta }}}_{p}){y}_{2}^{c}-4{g}^{3}\hslash {a}_{0}^{2}{X}_{0}\frac{{f}_{3}(2{{\rm{\Delta }}}_{p})}{m{f}_{2}(2{{\rm{\Delta }}}_{p})}{({A}_{1}^{+})}^{\ast }{X}_{1}\\  &  & +\,{A}_{1}^{-}{X}_{1}[4\alpha {g}^{2}{X}_{0}{\omega }_{m}^{2}\frac{{f}_{3}(2{{\rm{\Delta }}}_{p})}{{f}_{2}(2{{\rm{\Delta }}}_{p})}-iG\zeta (2{{\rm{\Delta }}}_{p})]\}.\end{array}$$

For the amplitudes of the first-order upper and lower sidebands in Eqs (20) and (21), the first term is the contribution of the probe pulse, while the other term is relevant to the coherent mechanical pump with the two-phonon process. As expected, the first-order sidebands are proportional to both probe pulse and mechanical pump amplitudes, whereas the second-order sidebands exhibit a complex frequency-conversion via the photon-phonon and phonon-phonon interactions.

Subsequently, we focus on the output-light fields that transmit through the left mirror of the cavity. Associating with the input-output relation of cavity, we have the output transmission spectrum as follows:

23 $${S}_{out}=\sqrt{{\eta }_{L}\kappa }a-{S}_{in}^{I}={c}_{1}+{c}_{p}{e}^{-i{{\rm{\Delta }}}_{p}t}+\sqrt{{\eta }_{L}\kappa }{A}_{1}^{+}{e}^{i{{\rm{\Delta }}}_{p}t}+\sqrt{{\eta }_{L}\kappa }\sum _{z=2}^{n}({A}_{z}^{-}{e}^{-zi{{\rm{\Delta }}}_{p}t}+{A}_{z}^{+}{e}^{zi{{\rm{\Delta }}}_{p}t}),$$

with ${c}_{1}=\sqrt{{\eta }_{c}\kappa }{\alpha }_{s}-{\varepsilon }_{c}$ and ${c}_{p}=\sqrt{{\eta }_{c}\kappa }{A}_{1}^{-}-{\varepsilon }_{p}$. ${S}_{in}^{I}$ is the transformation form of *S*_*in*_ in a rotating frame of control field frequency *ω*_*c*_. The transmission of probe pulse is defined as ${t}_{p}={c}_{p}/{\varepsilon }_{p}{e}^{-i{\varphi }_{pc}}$ that can be used to study the first-order upper sideband and the two-phonon optomechanically induced transparency. And, the ${\eta }_{n}=|\sqrt{{\eta }_{c}\kappa }{A}_{n}^{-}/{\varepsilon }_{p}|$ refers to the amplitude of the *n* ^*th*^-order upper sideband, in which the amplitude of probe pulse is treated as a basic scale to gauge the amplitude of the output sideband *η*_*n*_. For example, *η*_*n*_ = 0.2 means that the amplitude value of *n* ^*th*^-order sideband is equal to 0.2 times of probe pulse amplitude, rather than that 0.2 times of probe pulse amplitude are converted into the *n* ^*th*^-order sideband. Besides, the amplitude ratio between mechanical pump and probe pulse is defined as *n*_0_ = *ε*_*d*_/*ε*_*p*_. Due to the complexity of phase superposition in these high order nonlinear processes, for simplicity, we assume the relative phase of probe pulse is zero, i.e., *ϕ*_*pc*_ = 0.

In above mathematical derivation of higher-order sidebands, there are four assumptions used to simplify the numerical results. In order to verify their validity, we give a clear description for these assumptions, as follows:

1. *The approximation of quadratic optomechanical coupling in membrane-in-the-middle optomechanical structure*. For our proposed optomechanical system, wherever the membrane is, the complete Hamiltonian can be expressed as
  24 $$\begin{array}{rcl}H & = & \frac{{\hat{p}}^{2}}{2m}+\frac{1}{2}m{\omega }_{m}{\hat{x}}^{2}+i\hslash {\varepsilon }_{d}[{({\hat{b}}^{\dagger })}^{2}{e}^{-i{\omega }_{d}t-i{\varphi }_{d}}-{\hat{b}}^{2}{e}^{i{\omega }_{d}t+i{\varphi }_{d}}]+\hslash \omega (\hat{x}){\hat{a}}^{\dagger }\hat{a}\\  &  & +\,i\hslash \sqrt{{\eta }_{L}\kappa }{\varepsilon }_{c}({\hat{a}}^{\dagger }{e}^{-i{\omega }_{c}t}-\hat{a}{e}^{i{\omega }_{c}t})+i\hslash \sqrt{{\eta }_{L}\kappa }{\varepsilon }_{p}({\hat{a}}^{\dagger }{e}^{-i{\omega }_{p}t-i{\varphi }_{pc}}-\hat{a}{e}^{i{\omega }_{p}t+i{\varphi }_{pc}}),\end{array}$$
  here the exact cavity frequency is defined as $\omega (x)={\omega }_{q}+\frac{\pi }{\tau }-\frac{1}{\tau }[{\sin }^{-1}(\sqrt{R}\,\cos \,2{k}_{q}x)+{\sin }^{-1}(\sqrt{R})]$ (with *k*_*q*_ = *ω*_*q*_/*c* and *τ* = *L*/*c*), so that there is an odd number of half wavelengths in the whole cavity. In the simple case of *R* = 1 and *x* = 0, the resonant frequencies of the two subcavities are ${\omega }_{q}=\frac{q\pi c}{L}$(with *q* = 2*L/λ*_*q*_, *λ*_*q*_ = 2*πc*/*ω*_*q*_ and the cavity-mode number *q*). If the membrane is located at an antinode of the frequency *ω*(*x*) of cavity field, the approximate cavity frequency can be expressed as the second order of *x*, i.e., $\omega (x)={\omega }_{0}+\frac{1}{2}\frac{{d}^{2}\omega }{d{x}^{2}}{|}_{x=0}{x}^{2}$. Then, we assume that the quadratic coupling constant is defined as $G=\frac{1}{2}\frac{{d}^{2}\omega }{d{x}^{2}}{|}_{x=0}=\frac{8{\pi }^{2}c}{L{\lambda }_{c}^{2}}\sqrt{\frac{R}{1-R}}$ ^25,29^. By substituting the approximate cavity frequency into Eq. (24), we can obtain the system’s Hamiltonian Eq. (1) of this dispersive optomechanical cavity. Under this approximation of quadratic optomechanical coupling, we discuss conveniently the two-photon sideband effect.
2. *Sideband-resolved limit* ${\omega }_{m}\gg \kappa$ *in cavity optomechanics*. It is well known that, in mechanical effects of light, *κ* ^−1^ refers to the lifetime of cavity field. And the frequency difference between *ω*_*c*_ and *ω*_*p*_ in output spectrum lines of cavity field equals to *ω*_*m*_. These spectrum lines can be well distinguished only when ${\omega }_{m}\gg \kappa$. Such a parameter limit is called sideband-resolved limit. Under this approximation of sideband-resolved limit, the studies for various optomechanical phenomena including sideband cooling^5–7^ and higher-order sideband generation^16–20^ have a reality-based physical meaning.
3. *The factorization assumption of* 〈*ab*〉 = 〈*a*〉〈*b*〉. As we all know, all of the observable quantities are confined by uncertainty principle in the field of quantum mechanics. However, in our proposed optomechanical system, the order of magnitude of both input coherence light and the output fields can reach microwatt, so that these optical fields can be regarded as the statistical result of photons. In this case, the uncertainty principle of quantum theory is replaced by the classical limit, in which the Planck constant is treated as zero (i.e., ħ → 0) and the expectation values of physical quantities satisfies the factorization 〈*ab*〉 = 〈*a*〉〈*b*〉. Because the input and output optical fields in the form of coherent light interact with the quadratically coupled optomechanical system, this factorization assumption is valid for the calculation of the expectation values of both cavity field and other quantities in Eqs (3)–(8).
4. *The perturbation theory*. In order to solve the Heisenberg-Langevin equations (3)–(8), the expectation values of the operators are required to have a perturbation form of ${\mathscr{O}}(t)={{\mathscr{O}}}_{0}+\delta {\mathscr{O}}(t)$, in which the steady-state value ${{\mathscr{O}}}_{0}$ is far more than the perturbation value $\delta {\mathscr{O}}(t)$. In our proposed quadratically coupled optomechanical model, the control field intensity is much stronger than the intensities of probe pulse and mechanical pump. In this optomechanical environment, the control field is used to excite the cavity field and support the steady-state value, while the probe pulse and mechanical pump participate in the generation of higher-order sidebands. It should be pointed out that the theoretical essence of this perturbation method is the same as Van Vleck perturbation theory^40,41^. The difference of these two perturbation theory is the calculation process. In detail, Van Vleck perturbation theory is directly used to solve the Schrödinger equation due to the predictable wave functions or energy eigenvalues of the system, while the perturbation method in the present paper is used to solve the Heisenberg-Langevin equations by expanding the perturbation terms in a form of Fourier series.

Before discussing the higher-order sideband generation with the multiphonon processes, the experimental feasibility of this quadratically coupled optomechanical model should be introduced. According to a realistic parameter set of recent experiment in cavity-optomecheanics system^22^, the membrane is movable with the angular frequency *ω*_*m*_ = 2*π* × 0.1 *MHz*, the mass *m* = 100 *pg*, and the mechanical quality factor *Q* = *ω*_*m*_/Γ_*m*_ = *π* × 10^4^. And the membrane’s reflectivity determined by the fraction of intracavity photons that transfer momentum to the membrane is chose to be *R* = 0.8. In addition, we assume the cavity length *L* = 67 *mm*, the total loss rate of cavity field *κ* = 0.2*ω*_*m*_ and the wave length of control field *λ*_*c*_ = 2*πc*/*ω*_*c*_ = 532 *nm*. In this scenario, the cavity mode detuning is assumed to be Δ_*c*_ = 2*ω*_*m*_ for building a two-phonon resonance case. Note that unlike the probe transmission spectrum transmitted through the right mirror of cavity in ref.^27^, our result will have an inverse transmission spectrum due to the output terminal transmitting through the left mirror of cavity.

## Numerical Results and Discussions

**Figure 2:**
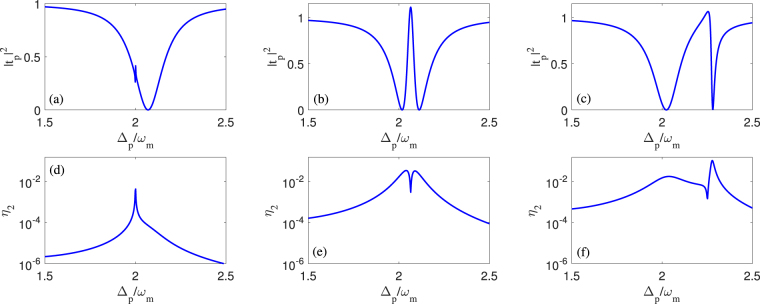
The transmission intensity of probe pulse |*t*_*p*_|^2^ and the amplitude of second-order upper sideband *η*_2_ versus the probe-pulsed detuning Δ_*p*_ for different control field intensities. (i) we use *P*_*c*_ = 0.1 *μW* in panels (a) and (d), (ii) we use *P*_*c*_ = 50 *μW* in panels (b) and (e), and (iii) we use *P*_*c*_ = 200 *μW* in panels (c) and (f). Other parameters are *m* = 100 *pg*, *ω*_*m*_ = 2*π* × 0.1 *MHz*, *Q* = *ω*_*m*_/Γ_*m*_ = *π* × 10^4^, *L* = 67 *mm*, *T* = 50 *K*, *κ* = 0.2*ω*_*m*_, Δ_*c*_ = 2*ω*_*m*_, *η*_*L*_ = *η*_*R*_ = 0.499, *ε*_*p*_ = 0.05*ε*_*c*_, and *n*_0_ = 0.001.

**Figure 3:**
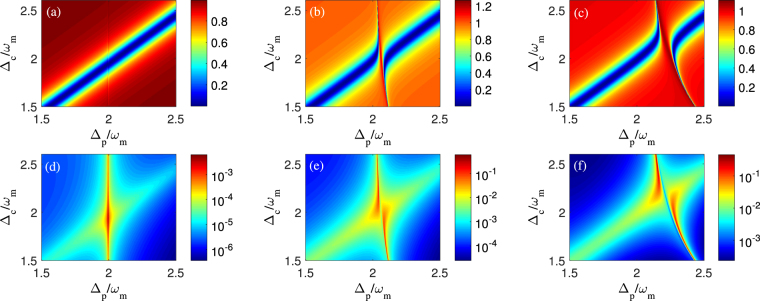
Contour maps of the transmission intensity of probe pulse |*t*_*p*_|^2^ (including (**a**–**c**) and the logarithm of amplitude *η*_2_ of second-order upper sideband (including (**d**–**f**) as a function of the probe-pulsed detuning Δ_*p*_ and the control field detuning Δ_*c*_ with different control field intensities. (i) we use *P*_*c*_ = 0.1 *μW* in panels (a) and (d), (ii) we use *P*_*c*_ = 50 *μW* in panels (b) and (e), and (iii) we use *P*_*c*_ = 200 *μW* in panels (c) and (f). Other parameters are the same as in Fig. 2.

**Figure 4:**
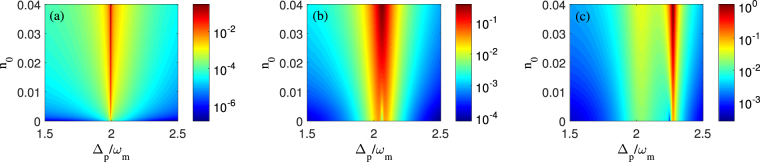
The logarithm of the amplitude *η*_2_ of second-order upper sideband as a function of the probe-pulsed detuning Δ_*p*_ and the amplitude ratio *n*_0_ with different control fields. (**a**) *P*_*c*_ = 0.1 *μW*, (**b**) *P*_*c*_ = 50 *μW*, and (**c**) *P*_*c*_ = 200 *μW*. Other parameters are the same as in Fig. 2.

**Figure 5:**
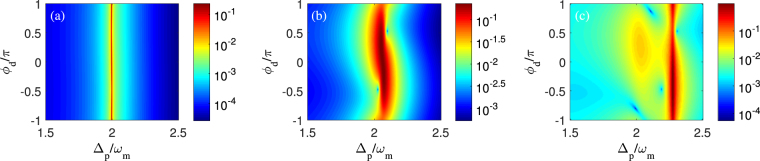
The logarithm of the amplitude *η*_2_ of second-order upper sideband as a function of the probe-pulsed detuning Δ_*p*_ and the relative phase *ϕ*_*d*_ with different control field intensities. (**a**) *P*_*c*_ = 0.1 *μW*, (**b**) *P*_*c*_ = 50 *μW*, and (**c**) *P*_*c*_ = 200 *μW*. Other parameters are the same as in Fig. 2 except for *n*_0_ = 0.03.

**Figure 6:**
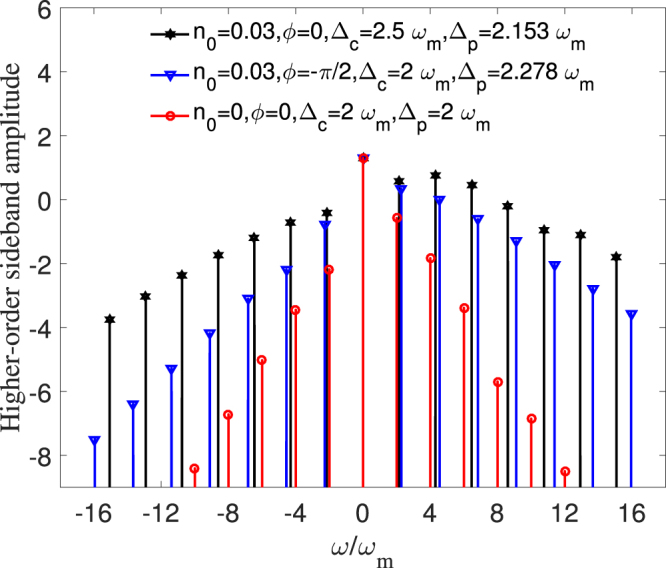
The output higher-order sideband spectra (in logarithmic scale). Other parameters are are the same as in Fig. 2(f).

**Figure 7:**
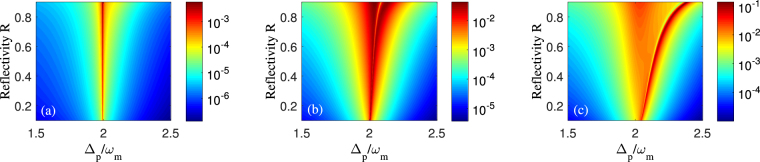
The logarithm of the amplitude *η*_2_ of second-order upper sideband as a function of the reflectivity of membrane *R* and the probe-pulsed detuning Δ_*p*_ with different control fields. (**a**) *P*_*c*_ = 0.1 *μW*, (**b**) *P*_*c*_ = 50 *μW*, and (**c**) *P*_*c*_ = 200 *μW*. Other parameters are the same as in Fig. 2.

In this section, we firstly focus on the properties of the second-order sideband based on the analytical expressions (20)–(22). In this situation, we analyze the influences of the system parameters, including the intensity and frequency detuning of control field, as well as the amplitude and phase of the mechanical pump in Figs 2–5. There are two additional remarks for the parameter choice of probe pulse. 1. We assume that the amplitude of probe pulse is proportional to that of control field based on the relationship of *ε*_*p*_ = 0.05*ε*_*c*_. 2. The numerical results of higher-order sidebands are normalized by the definition of ${\eta }_{n}=|\sqrt{{\eta }_{c}\kappa }{A}_{n}^{-}/{\varepsilon }_{p}|$, which is dimensionless. Whatever the intensity of probe pulse is how to change, the higher-order sideband signal can be regarded as amplification when the normalized amplitudes *η*_*n*_ have an enhancement. Next, according to Eq. (23), we turn to illustrate the normalized amplitudes of output higher-order sidebands by the numerical simulations of |*S*_*out*_/*ε*_*p*_| in frequency domain (see Fig. 6 that includes seven orders of sidebands). It should be noted that the analytical expressions of first- and second-order sideband amplitudes are shown in Eqs (20)–(22), while the *n* ^*th*^-order sideband amplitudes ${A}_{n}^{-}$ and ${A}_{n}^{+}$ (*n* > 2) can be obtained from the derivation of low-order sidebands. However, the *n* ^*th*^-order sideband amplitudes ${A}_{n}^{-}$ and ${A}_{n}^{+}$ (*n* > 2) do not exhibit in this paper due to their complex form of analytical expressions. Finally, we also give some discussion about the influence of the membrane reflectivity on second-order sideband in Fig. 7.

Within above practical parameter set, first of all we analyze the optical properties of the probe-pulsed transmission and the second-order upper sideband for an optomechanical circumstance with a very small amplitude ratio between mechanical pump and probe pulse, i.e., *n*_0_ = 0.001. Therefore, Fig. 2 shows the transmission intensity |*t*_*p*_|^2^ of probe pulse and the amplitude *η*_2_ of second-order upper sideband versus the probe-pulsed detuning Δ_*p*_ for three different control field intensities: (i) *P*_*c*_ = 0.1 *μW*, (ii) *P*_*c*_ = 50 *μW* and (iii) *P*_*c*_ = 200 *μW*. When the control field is relatively weak, i.e., *P*_*c*_ = 0.1 *μW*, one can find that, from Fig. 2(a), the probe transmission spectrum experiences an opacity dip near the two-phonon resonance condition Δ_*p*_ ≈ 2*ω*_*m*_, where the probe pulse is almost completely absorbed (this probe resonance absorption is called non-OMIT in the following discussion). If the intensity of control field increases from 0.1*μW* to 50*μW* in Fig. 2(b), there is a standard two-phonon OMIT described by an obvious transparency window between two symmetric opacity dips, which results from the quantum destructive interference^25–27^. As the intensity of control field continues to increase and reaches 200 *μW*, an asymmetric lineshape of two-phonon OMIT, that is characterized by a smooth dip within broad frequency ranges and a sharp dip within extremely narrow frequency ranges^42–44^, appears in the probe transmission spectrum; see Fig. 2(c). Direct comparison these three probe transmission spectra exhibits that the opacity dip in Fig. 2(a), the transparency window in Fig. 2(b) and the asymmetric dip in Fig. 2(c) are respectively located at different detunings of effective cavity resonance frequency, i.e., ${{\rm{\Delta }}}_{p}\approx {\bar{{\rm{\Delta }}}}_{c}\approx 2{\omega }_{m}$, ${{\rm{\Delta }}}_{p}\approx {\bar{{\rm{\Delta }}}}_{c}\approx 2.065{\omega }_{m}$ and ${{\rm{\Delta }}}_{p}\approx {\bar{{\rm{\Delta }}}}_{c}\approx 2.278{\omega }_{m}$. On the other hand, associating |*t*_*p*_|^2^ in Fig. 2(a–c) with *η*_2_ in Fig. 2(d–f), it can be seen that the amplitudes of second-order upper sidebands coincide inversely with the line shapes of probe transmission spectra. Such a high-consistency between |*t*_*p*_|^2^ and *η*_2_ comes from the nonlinear parametric frequency-conversion where the probe pulse is actually depleted to support the higher-order sideband generation. More interestingly, an enhanced second-order sideband can be achieved in the position of that asymmetric dip; see Fig. 2(f). In other words, the asymmetric two-phonon OMIT caused by the quadratic optomechanical coupling assisted by a strong control field and a weak probe pulse opens a high-efficiency channel for the second-order sideband generation.

Next, in order to build a tunable sideband amplification, we will study the dependence of higher-order sidebands on the system parameters of optomechanical cavity including the frequency detuning of control field, the amplitude and phase of the mechanical pump and the membrane reflectivity. Note that we focus on the properties of higher-order sidebands based on the above mentioned three optomechanical circumstances (i.e., non-OMIT in Fig. 2(a), standard two-phonon OMIT in Fig. 2(b) and asymmetric lineshape of two-phonon OMIT in Fig. 2(c)), which symbolize three optomechanical phenomena.

Now we start to evaluate the dependence of second-order sideband on the frequency detuning of control field. Physically, the detuning management of nonlinear response plays a supplementary role in the modulation of optical nonlinearity^45^. It means that the frequency detuning between cavity field and control field can modify the optical nonlinearity strength of optomechanical system and affect the amplitude of second-order sideband output. Therefore, we plot the transmission intensity of probe pulse |*t*_*p*_|^2^ (see Fig. 3(a–c)) and the amplitude of second-order upper sideband *η*_2_ (see Fig. 3(d–f)) varying with the probe-pulsed detuning Δ_*p*_ and the control field detuning Δ_*c*_ for three different control field intensities that are same with Fig. 2. For the three cases of *η*_2_ in Fig. 3(d–f), one can find that *η*_2_ depends sensitively on Δ_*c*_. In detail, the local maximum of *η*_2_ occurs at the position of Δ_*c*_ = 1.9*ω*_*m*_ when *P*_*c*_ = 0.1 *μW* in Fig. 3(d). With *P*_*c*_ increasing to 50 *μW* in Fig. 3(e) or 200 *μW* in Fig. 3(f), the local maximums of *η*_2_ have a giant enhancement at the off-resonance position of the control field, i.e., Δ_*c*_ ≠ 2*ω*_*m*_. Comparing |*t*_*p*_|^2^ in Fig. 3(a–c) with *η*_2_ in Fig. 3(d–f), it shows that, for a fixed control field detuning Δ_*c*_, the maximums of *η*_2_ are always located in the extremely narrow frequency ranges where their corresponding transmission intensities |*t*_*p*_|^2^ exhibit an asymmetric dip, just as illustrated in Fig. 2. This present result also confirms that the frequency detuning of control field allows us to modify the transmission of probe pulse and improve the amplitude of second-order sideband generation beyond what is achievable in the quadratically coupled optomechanical system based on the conventional linearized approximation.

Based on the analytical expression of the amplitude of second-order sideband in Eq. (22), it is readily found that both the amplitude and phase of coherent mechanical pump play an important role in the generation of multiphonon sideband effect in this quadratically coupled optomechanical system. Then, we proceed to numerically study the multiphonon sideband effect and focus on the sideband amplification and phase-sensitive dependence by adjusting the amplitude and phase of coherent mechanical pump. Because of the high-consistency between |*t*_*p*_|^2^ and *η*_2_, now we turn to only investigate the second-order sideband in the following discuss.

In Fig. 4, we plot the amplitude *η*_2_ of second-order upper sideband as a function of the probe-pulsed detuning Δ_*p*_ and the amplitude ratio *n*_0_ for three different optomechanical circumstances, i.e., non-OMIT, standard two-phonon OMIT and asymmetric lineshape of two-phonon OMIT, which have been introduced in Fig. 2. Firstly, by making a qualitative analysis for Fig. 4, it is clear that, with the amplitude ratio *n*_0_ increasing, *η*_2_ in Fig. 4(a) always keeps a emission peak at the two-phonon resonance frequency Δ_*p*_ ≈ 2*ω*_*m*_. Whereas an opacity dip of *η*_2_ in both Fig. 4(b) and (c) dramatically evolves into the only strong emission peak as the amplitude ratio *n*_0_ increases. Similar to the microwave field applied to the three-level atomic^46,47^, the use of additional mechanical pump has been explored the parametric amplification and phase-sensitive dependent^48,49^. Secondly, through a quantitative analysis for the three cases with different control field intensities in Fig. 4(a–c), one can see that the amplitudes of second-order sideband can be significantly enhanced, even approach to the probe pulse intensity in Fig. 4(c), by increasing either the intensity of control field or mechanical pump. And, whatever *n*_0_ is how to vary, the maximums of *η*_2_ for these three cases are located at the same effective cavity resonance frequency, i.e., Δ_*p*_ ≈ 2*ω*_*m*_ in Fig. 4(a), Δ_*p*_ ≈ 2.065*ω*_*m*_ in Fig. 4(b) and Δ_*p*_ ≈ 2.278*ω*_*m*_ in Fig. 4(c). This phenomenon can be also explained by the perturbation theory. Under the condition of the fixed cavity mode *ω*_0_, the effective cavity resonance frequency *ω*_0_ + *GX*_0_ is proportional to the square value of displacement of the membrane *X*_0_, while *X*_0_ displayed in Eq. (10) only depends on the intensity of control field. Correspondingly, the position of maximum *η*_2_ is determined by the intensity of control field rather than the other perturbation terms including the probe pulse and the mechanical pump. It’s worth noting that such a mechanical pump could be tuned by using microwave electrical driven^50^ and other time-varying weak forces. As a result, we would provide a novel method to amplify the second-order sideband generation in a convenient way.

For give a better insight on the phase-dependent effect for the generated second-order upper sideband, in Fig. 5, we plot the amplitude *η*_2_ of second-order sideband versus the probe-pulsed detuning Δ_*p*_ and the relative phase *ϕ*_*d*_ of mechanical pump with three different control field intensities. Because the relative phase *ϕ*_*d*_ is attached in the mechanical pump, we choose a relatively large power of mechanical pump, i.e., *n*_0_ = 0.03, to improve the phase sensitivity. When we adopt a weak control field *P*_*c*_ = 0.1 *μW* to ensure non-OMIT effect in Fig. 5(a), the phase-dependent effect of *η*_2_ begins to occur at the two-phonon resonance frequency Δ_*p*_ ≈ 2*ω*_*m*_. If the control field becomes strong, the phase-dependent effect has a significant influence on the second-order sideband spectra, in which the phase-dependent effect exhibits an odd symmetry with respect to (2.065*ω*_*m*_,0) in Fig. 5(b) and an asymmetric phase-dependent effect emerges in Fig. 5(c). Moreover, the maximum *η*_2_ is still located at the respective cavity resonance frequency, which agrees with the results of Fig. 4. Physically, the phase-sensitive dependence of the nonlinear frequency-conversion, involving photon-phonon and multiphonon processes, relies on the degree of phase mismatching accumulated by parametric up-convert or down-convert paths that lead to the destructive or constructive quantum interference.

Up to now, we have demonstrated that the control and amplification for generated second-order sideband can be achieved in this quadratically coupled optomechanical system assisted by a strong control field, a weak probe pulse and an external mechanical pump. Then, a natural question is whether or not these fascinating features apply to the total higher-order sidebands? To making an intuitional picture that contains a full output transmission spectrum, we plot the two-phonon higher-order sideband spectra with different parameters as shown in Fig. 6. Here, these fixed probe-pulsed detunings are chose in the position of a series of maximum second-order sideband amplitudes, which can be obtained in Figs 3 and 4. In the absence of the coherent mechanical pump, i.e., *n*_0_ = 0, the red spectrum line shows that the amplitude of higher-order sidebands decreases rapidly as the order number increases. When a strong mechanical pump is applied to the optomechanical system, i.e., *n*_0_ = 0.03, it can be seen clearly that the amplitudes of both blue spectrum line and black spectrum line decrease slowly with the increase of the order number, leading to a broad platform of multiphonon sidebands. Due to the detuning management of nonlinearity acquired by Δ_*c*_, a more robust higher-order sideband spectrum occurs when Δ_*c*_ = 2.5*ω*_*m*_; see the black spectrum line in Fig. 6. In comparison with three spectrum lines of Fig. 6, it implies that the two-phonon higher-order sideband spectrum can be simultaneously controlled and amplified by modulating both the mechanical driving field and the control field detuning. From application point of view, such an enhanced higher-order sideband proposal provides a practical opportunity to implement chip-scale optical communications and optical frequency combs.

Last but not least, we also consider the influence of the membrane reflectivity on the second-order sideband. From the expression of quadratic coupling constant $G=\frac{1}{2}\frac{{d}^{2}\omega }{d{x}^{2}}{|}_{x=0}=\frac{8{\pi }^{2}c}{L{\lambda }_{c}^{2}}\sqrt{\frac{R}{1-R}}$ ^25,29^, we find that *G* increases with the membrane reflectivity *R* increasing. Correspondingly, the amplitude of second-order upper sideband *η*_2_ varying with the membrane reflectivity *R* and the probe-pulsed detuning Δ_*p*_ is shown in Fig. 7. There are two common characteristics for the three cases of different control field intensities in Fig. 7(a–c) and (i) With *R* increasing, the amplitude of *η*_2_ is expected to be enhanced due to the increase of the quadratic optomechanical coupling and the strengthen photon-phonon interaction between the optical and mechanical modes. (ii) It can be seen that, with *R* increasing, the spectrum width of the emission peak or the opacity dip becomes more and more broad. In comparison with the low membrane reflectivity applied in this quadratically coupled optomechanical system, the use of the high membrane reflectivity can not produce a new feature for the sideband effects, but make the original sidebands more obvious. That is, the second-order sideband effect can’t be confined by the change of the membrane reflectivity, which is favorable from viewpoint of the experiments.

## Experimental realization of our proposed scheme

Before making a conclusion, we give a concise description about the experimental feasibility of our proposed scheme. Although the sufficient optomechanical coupling of mechanical devices reaching the quantum regime has been a outstanding technical challenge, the strong and tunable dispersive optomechanical coupling was reported in high-finesse Fabry-Pérot cavity with good mechanical properties (high *Q*; small *m*, spring constant *k*)^22–24^. In these works, the quadratic optomechanical coupling is increased several orders of magnitude beyond previous devices, while the linear optomechanical coupling vanishes. According to the experimental achievements of ref.^22,24^ a *SiN* membrane (1 *mm* × 1 *mm* × 50 *nm*) on a silicon chip is mounted to the waist of the cavity field. In detail, when the membrane is placed at an antinode of cavity field, the cavity finesse is set as *F*_*AN*_ = 6940, while the finesse *F*_*N*_ = 15200 corresponds to the membrane position at a node. By maintaining such a high finesse, the mechanical device is not heated by absorption of light. This dispersive optomechanical device with the high finesse has an experimental repeatability even when the membrane is precisely placed at a node or antinode of the cavity field^22^. More importantly, one advantage in the quadratically coupled optomechanical system is the more accessible quantum behaviors, such as the cooling of the membrane from the staring temperature of 294 *K* ^22^, the delay and store of classical light pulses^26^. It should be emphasized that the two-phonon higher-order sideband scheme must be realized at the low temperature environment, because the present numerical results involve the factorization assumption 〈*ab*〉 = 〈*a*〉〈*b*〉 that indicates the boundary between quantum and classical physics. We believe that our proposed quadratically coupled optomechanical system can be also realized by the existing experimental techniques of optomechanical cavity with micro-structured materials.

## Conclusion

In conclusion, we have performed a theoretical analysis for the controllable amplification of two-phonon higher-order sidebands in the quadratically coupled optomechanical system, where the optical cavity mode couples quadratically rather than linearly to the position of a membrane. Beyond the conventional linearized approximation, the nonlinear terms are added into the Heisenberg-Langevin formalism. Thus, we derive analytical expressions for the output transmission of probe pulse and the amplitude of second-order sideband based on the perturbation technique. With the help of quadratic coupling between the optical and mechanical modes, we show that the mechanical pump and the frequency detuning of control field allow us to modify the output transmission of probe pulse and amplify the two-phonon higher-order sidebands. Comparing with the previous schemes in linear coupled optomechanical system^16,48^, the maximum amplitude of second-order sideband, for a suitable designed quadratically coupled optomechanical system, can approach to the probe pulse amplitude. We also reveal that the higher-order sideband generation depends sensitively on the phase of the mechanical pump when the control field becomes strong. Furthermore, the present results illustrate the potential to utilize quadratic optomechanical coupling for optimizing the two-phonon higher-order sidebands, as well as a guidance in the design of chip-scale optical communications and optical frequency combs.
